# Effect of mobile health reminders on tuberculosis treatment outcomes in Shanghai, China: A prospective cohort study

**DOI:** 10.3389/fpubh.2023.923319

**Published:** 2023-04-27

**Authors:** Zheyuan Wu, Liping Lu, Yong Li, Jing Chen, Zurong Zhang, Chenxi Ning, Zheng’an Yuan, Qichao Pan, Xin Shen, Wenhong Zhang

**Affiliations:** ^1^Department of Infectious Diseases, Huashan Hospital, Fudan University, Shanghai, China; ^2^Department of Tuberculosis Control, Shanghai Municipal Center for Disease Control and Prevention, Shanghai, China; ^3^Shanghai Institutes of Preventive Medicine, Shanghai, China; ^4^Songjiang District Center for Disease Control and Prevention, Shanghai, China; ^5^Shanghai Municipal Center for Disease Control and Prevention, Shanghai, China

**Keywords:** digital health, mobile application, medication monitor, tuberculosis, treatment outcome

## Abstract

**Background:**

Poor adherence increases the risk of unfavorable outcomes for tuberculosis (TB) patients. Mobile health (mHealth) reminders become promising approaches to support TB patients’ treatment. But their effects on TB treatment outcomes remain controversial. In this prospective cohort study, we evaluated the effect of the reminder application (app) and the smart pillbox on TB treatment outcomes compared with the standard care in Shanghai, China.

**Methods:**

We recruited new pulmonary TB (PTB) patients diagnosed between April and November 2019 who were aged 18 or above, treated with the first-line regimen (2HREZ/4HR), and registered at Songjiang CDC (Shanghai). All eligible patients were invited to choose the standard care, the reminder app, or the smart pillbox to support their treatment. Cox proportional hazard model was fitted to assess the effect of mHealth reminders on treatment success.

**Results:**

260 of 324 eligible patients enrolled with 88 using standard care, 82 the reminder app, and 90 the smart pillbox, followed for a total of 77,430 days. 175 (67.3%) participants were male. The median age was 32 (interquartile range [IQR] 25 to 50) years. A total of 44,785 doses were scheduled for 172 patients in the mHealth reminder groups during the study period. 44,604 (99.6%) doses were taken with 39,280 (87.7%) monitored by the mHealth reminders. A significant time-dependent downward linear trend was observed in the monthly proportion of dose intake (*p* < 0.001). 247 (95%) patients were successfully treated. The median treatment duration of successfully treated patients in the standard care group was 360 (IQR 283–369) days, significantly longer than those in the reminder app group (296, IQR 204–365, days) and the smart pillbox group (280, IQR 198–365, days) (both *p* < 0.01). Using the reminder app and the smart pillbox was associated with 1.58 times and 1.63 times increase in the possibility of treatment success compared with the standard care, respectively (both *p* < 0.01).

**Conclusion:**

The reminder app and the smart pillbox interventions were acceptable and improved the treatment outcomes compared with the standard care under the programmatic setting in Shanghai, China. More high-level evidence is expected to confirm the effect of mHealth reminders on TB treatment outcomes.

## Introduction

Tuberculosis (TB) remains one of the leading causes of death worldwide (1). The treatment success rate for TB patients treated with first-line regimens was steadily around 85% in recent years, globally (1). However, the standard first-line regimen requires people to take 2 to 4 medicines daily for at least 6 months (2). Poor adherence to anti-TB chemotherapy increases the risks of morbidity, mortality, drug resistance and results in unfavorable outcomes (3). The World Health Organization (WHO) has recommended directly observed therapy (DOT) for decades to promote adherence to TB treatment. DOT significantly increased the rates of treatment success, adherence, sputum smear conversion and lowered the rate of development of drug resistance when compared with self-administered therapy (SAT) (4). But it’s labor-intensive for both patients and healthcare providers (5), which makes it unfeasible to cover all patients in high TB burden countries.

China accounted for 8.5% of the 9.9 million estimated incident TB cases in 2020, ranking second in the world (1). Although the TB prevalence has been halved due to the implementation of the DOTS strategy from 1990 to 2010 (6), treatment adherence interventions are still challenging. A systematic review revealed that 52% of TB patients were on SAT, and 28% were observed by family members (7). In Shanghai, TB patients are managed by doctors from community health centers (CHC) (8). Due to the limit of health care resources, they usually train a family member to conduct DOT and check off the medication calendar every day. They visit patients regularly to evaluate their adherence and offer medical support. Therefore, it is hard for them to discover the non-adherence and take essential interventions in time. The increase of migrants and the aging population makes patient management more challenging since they often live alone and no qualified observers could be sought, indicating the urgent need for innovative adherence interventions to complement DOT.

WHO recently encouraged using digital health technologies such as short message service (SMS), video-supported treatment (VOT), and medication event monitoring systems (MEMS) to help TB patients complete treatment (2, 9). In China, an electronic medication monitor (EMM) without real-time data transmission has been used in 138 counties in three provinces (10). A cluster-randomized trial proved that the EMM improved medication adherence in TB patients while SMS did not (11). But the effect of the EMM and SMS on TB treatment outcomes remains controversial (10, 12–15). Additionally, the EMM cannot provide real-time medication adherence data which may impede timely intervention from healthcare providers.

With the rapid popularization of mobile technology, mobile health (mHealth) interventions such as reminder apps and devices with access to mobile phone networks become promising approaches to improve treatment adherence (16–18). We implemented a mHealth reminder system for TB that integrated the reminder application (app), the smart pillbox, and the management app/website to support TB patients’ treatment in Songjiang, Shanghai since 2018.

In this prospective cohort study, we aimed to evaluate the real-world effect of two mHealth reminders, i.e., the reminder app and the smart pillbox, on TB treatment outcomes compared with the standard care in Shanghai, China.

## Materials and methods

### Study setting and population

Shanghai is a 24-million-population metropolitan in China with a high proportion of internal migrants and an aging population. Internal migrants are those who migrate from their hometown to Shanghai, without a Shanghai household registration status. In Songjiang District, internal migrants accounted for 60% of the population in 2019, and 29% of residents aged 60 and above (19). TB patients were diagnosed in designated TB hospitals as per the provincial TB control program described before (20). Drug-susceptible TB patients were usually given the standard first-line regimen (2HREZ/4HR, isoniazid, rifampin, ethambutol, and pyrazinamide for 2 months, followed by isoniazid and rifampin for 4 months) at the beginning of treatment, and ordered to visit the TB hospital monthly to get medicine and conduct monitoring. Attending doctors evaluated the clinical efficacy at the end of the sixth month based on radiological and bacteriological data. The duration of anti-TB therapy was commonly extended month by month if the clinical efficacy was not favorable as the judgment of doctors. The treatment duration was typically no more than 1 year and the outcome was determined at the end of treatment by doctors. All pulmonary TB (PTB) patients living in Songjiang were mandatorily registered at Songjiang District Center for Diseases Control and Prevention (CDC) *via* a provincial TB management information system (TBMIS). Sixteen CHCs in Songjiang were in charge of the management of anti-TB treatment and also the care for TB patients under the supervision of Songjiang CDC. Residents, as well as migrants with Shanghai Residency Card, were reimbursed for the out-of-pocket expense (i.e., uncovered by medical insurance) of anti-TB drugs and essential examinations (e.g., chest X-ray, sputum bacteriological tests, blood biochemistry) at about 3,000 RMB ($474) on average.

### Study design

We conducted a prospective cohort study with the standard care group, the reminder app group, and the smart pillbox group in Songjiang, Shanghai. Newly diagnosed PTB patients between April and November 2019 were consecutively recruited if they were aged 18 or above, treated with the first-line regimen (2HREZ/4HR), and registered at Songjiang CDC. Patients with nontuberculous mycobacterial disease, rifampicin-resistant TB, HIV/AIDS, malignant tumors, renal failure, liver cirrhosis, mental diseases, and communication disorders were excluded. All sixteen CHCs in Songjiang participated in this study. All eligible patients were invited to participate and voluntarily choose one group to support their treatment. The use of the smart pillbox was completely free of charge. Patients choosing the reminder app group required that they or one of their family members owned smartphones with an internet connection paid for by themselves.

### The mHealth reminder system

**Figure 1:**
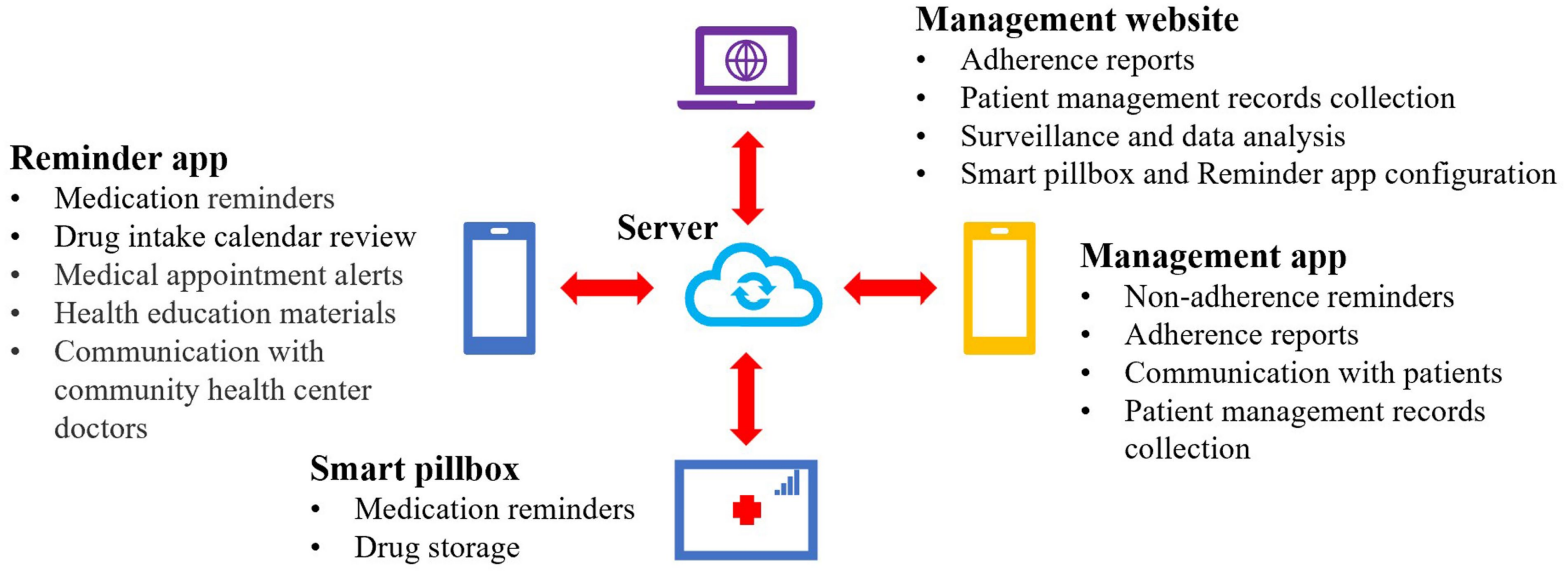
Schematic diagram for the mHealth reminder system. mHealth: mobile health.

The mHealth reminder system was developed by Beijing SINOVO POWER Technology Company (China) with in-depth customization by Shanghai Municipal CDC (Figure 1). The system provided two reminders for patients (i.e., the reminder app and the smart pillbox) and one management app as well as the management website for CHC doctors and CDC staff. Both of the apps were in Chinese and compatible with Android and iOS. TB patients could choose to use either of the two reminders but joint use was not allowed.

The smart pillbox was equipped with a 2G subscriber identity module card (SIM card) to connect to the internet, a chargeable battery to support 1-month use, and three LED lights with one beeper to indicate the work status and send reminders. It could contain four bottles of first-line drugs, which was the 1-month quantity (size: 18.5 cm × 12.5 cm × 6.5 cm). The pillbox could set at most three reminders a day using the management app or website. It beeped with the LED lights flashing at the scheduled time to remind patients to take medicine. If not opened, It would keep beeping and flashing for 1 min every 10 min in the following 30 min. Once opened, it stopped beeping and flashing and sent a signal to the server instantly to confirm the drug intake. If opened within 1 h before the scheduled time, a confirmation signal would be sent to the server and this reminder would not take effect. It would also remind patients if it was not closed properly or the battery was low. Its work status was transported to the server in real-time so that the CHC doctors could be informed and provide in-time help.

The reminder app could also set at most three reminders a day using the management app or website. It pushed drug intake alerts at the scheduled time every day and the patients tapped on the “confirm” button if they had taken the prescribed drugs. The alert would not be sent if patients had confirmed intake within 1 h before the scheduled time. It also provided functions of the drug intake calendar review, healthcare visit prompts, side effect reports, health education materials, patient forum, and communication with CHC doctors.

The management app pushed alerts late in the day (usually at 3 p.m.) if the patient had never opened the pillbox or confirmed drug intake in the app so that CHC doctors could take additional interventions. They could also check the real-time adherence reports, collect the management records, and conduct data analysis using the management app or by logging in to the management website to assist their supervision.

### Study procedures

Once PTB patients were diagnosed at the TB hospitals and registered at Songjiang CDC, the CHC doctors from the community where the patients lived would be informed instantly through the TBMIS and were required to meet the patients within 3 days to build the relationship and screen patients who met the enrolment criteria. The eligible patient was invited by CHC doctors to choose one study group to participate in. Enrolled patients were asked to complete a questionnaire about demographic information and followed for the whole treatment. The CHC doctors routinely appointed a family member as the treatment observer, who was trained to remind the patients to take daily medicine and visit the TB hospital monthly. Patients who lived alone were under self-administered treatment. The CHC doctors visited patients every 10 days during intensive phases and once a month during continuous phases to evaluate their adherence by checking medication calendars, medical records, and residual pills. They also offered medical support including instructions about adverse drug reactions, infection control, nutrition, and psychological problems. Patients were encouraged to contact CHC doctors at any time for help. CDC staff had no impact on the individual interventions.

### Standard care group

Patients in the standard care group were offered the standard care, which was a mix of family member observed treatment and self-administered treatment. The treatment observers conducted DOT and checked off the medication calendar every day. As for patients without observers, they took medicine and made records by themselves. The CHC doctors checked the medication calendar at every home visit and took essential interventions to improve adherence.

### Reminder app and smart pillbox groups

Patients in the reminder app and smart pillbox groups were trained to install and use the mHealth reminders by the CHC doctors after enrolling. The doctors set at least one drug intake reminder according to the patient’s prescriptions. Patients in the reminder app group were taught to tap on the “confirm” button after they had taken the drugs. Patients in the smart pillbox group were taught to store anti-TB medicine in the pillbox and take every dose from it. The CHC doctors would take interventions such as a phone call or a home visit to remind patients to take medicine and deal with medical or technical emergencies if they received intake missing alerts. They also retrained patients at every home visit if necessary.

### Data collection and statistical analysis

We collected demographic information using the questionnaire completed by the participants after enrollment. Clinical characteristics including the treatment outcomes were recorded by the TB hospital staff in the TBMIS and were extracted for analysis. Treatment outcomes followed the WHO definition (21). Treatment success was defined as the sum of cured and treatment completed. Treatment duration was defined as the time from the initial to the end of the anti-TB chemotherapy. Enrolled patients were followed until treatment outcomes occurred. Recurrence was determined by retrieval from TBMIS. Extrapulmonary TB (EPTB) was defined as TB involving organs other than the lungs. The majority of EPTB in the current study were tuberculous pleuritis. A bacteriologically confirmed case was defined as a PTB case whose biological specimen was positive smear microscopy, culture, or Xpert MTB/RIF.

Dose intake data for the patients in the reminder app and smart pillbox groups were extracted from the mHealth reminder system. The system labeled each scheduled dose during the treatment as “monitored by the reminders,” “recorded by the CHC doctors,” or “missed.” Once the pillbox was opened or the confirmation was made through the app, this dose was considered “monitored by the reminders.” If the CHC doctors confirmed that the patients had taken the dose but the system failed to record the data (either because of the system dysfunction or the patients’ failure to obey the procedure), they manually labeled this dose as “recorded by the CHC doctors.” Otherwise, the dose was labeled as “missed.”

Data analyses were on the intention-to-treat population. We compared categorical baseline characteristics and treatment outcomes between groups using Pearson’s Chi-squared test or Fisher’s exact test, as appropriate. Continuous characteristics were analyzed using Wilcoxon rank-sum test or Kruskal-Wallis rank-sum test followed by Dunn’s *post hoc* test for pairwise comparison. Dose intake data analysis was performed using the Cochran-Armitage trend test. We used Kaplan–Meier survival analysis to estimate the probability of treatment success and used the log-rank test to perform comparisons across study groups. We assessed the effect of mHealth reminders on treatment success using the Cox proportional hazard model. Unadjusted hazard ratios (HR) were calculated from univariate analysis. Adjusted hazard ratios were estimated by multivariate analysis. Factors in univariate analysis with a value of *p* less than 0.20 and other potentially associated ones entered the multivariate model. Power calculation for the comparison of survival curves under the Cox proportional hazard model was conducted using the “powerCT.default0” function of R package “powerSurvEpi.”

Analysis was performed using R^®^ software (4.1.2). A value of *p* less than 0.05 was considered significant.

### Ethical approval

The study was approved by the Ethical Review Committee at Shanghai Municipal Center for Disease Control and Prevention (2019-14). All enrolled patients provided written consent before inclusion in the study.

## Results

### Characteristics of the participants

**Figure 2:**
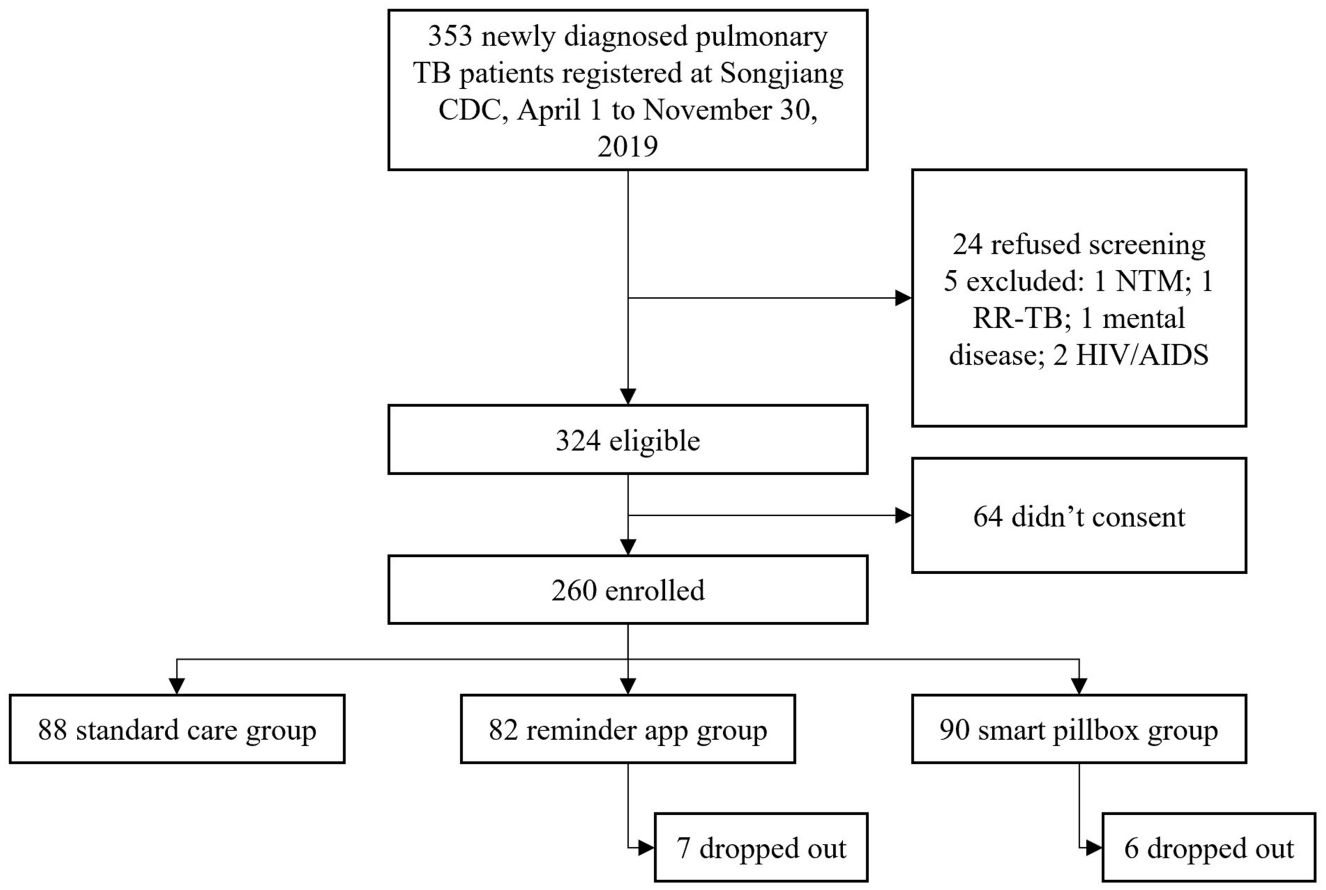
Flow chart. TB, tuberculosis; CDC, Center for Diseases Control and Prevention; NTM, nontuberculous mycobacterial disease; RR-TB, rifampicin-resistant tuberculosis.

From April 1 to November 30, 2019, 353 newly diagnosed PTB patients were registered at Songjiang CDC and 329 (93.2%) were successfully screened. Among 324 eligible patients, 260 (80.2%) enrolled with 88 in the standard care group, 82 in the reminder app group, and 90 in the smart pillbox group (Figure 2). The median training time was 20 [interquartile range (IQR) 10–30] and 10 (IQR 10–30) minutes for the reminder app group and the smart pillbox group, respectively (*p* = 0.002, Wilcoxon rank-sum test). 7 (8.5%) patients in the reminder app group and 6 (6.7%) in the smart pillbox group dropped out and continued treatment under the standard care. They were followed and retained in the initial study groups for the intention-to-treat analysis. 260 enrolled patients were followed for a total of 77,430 days (2,581 months) until treatment outcomes occurred, with no lost-to-follow-up.

Overall, 175 (67.3%) participants were male. The median age was 32 (IQR 25 to 50) years and differed significantly among the three groups (*p* < 0.001, Kruskal-Wallis test). Compared with the standard care group, the smart pillbox group was older (*p* = 0.005, Dunn’s test) and the reminder app group was younger (*p* < 0.001, Dunn’s test). The majority were migrants, married, students or employed, insured, covered by the reimbursement policy, and bacteriologically confirmed, with significant differences among the three groups (all *p* < 0.05; Table 1).

**Table 1:** Baseline characteristics of patients enrolled in the study.

Characteristic	Overall (*n* = 260)	Standard care (*n* = 88)	Reminder app (*n* = 82)	Smart pillbox (*n* = 90)	*p*-value^a^
Sex					0.761
Female	85 (32.7%)	28 (31.8%)	25 (30.5%)	32 (35.6%)	
Male	175 (67.3%)	60 (68.2%)	57 (69.5%)	58 (64.4%)	
Age, years					
Median (IQR)	32 (25, 50)	30 (26, 53)	26 (24, 33)	44 (31, 65)	<0.001
18–29	114 (43.8%)	42 (47.7%)	52 (63.4%)	20 (22.2%)	<0.001
30–44	65 (25.0%)	16 (18.2%)	22 (26.8%)	27 (30.0%)	
45–59	33 (12.7%)	14 (15.9%)	4 (4.9%)	15 (16.7%)	
> = 60	48 (18.5%)	16 (18.2%)	4 (4.9%)	28 (31.1%)	
Migrant					<0.001
No	86 (33.1%)	21 (23.9%)	16 (19.5%)	49 (54.4%)	
Yes	174 (66.9%)	67 (76.1%)	66 (80.5%)	41 (45.6%)	
Completed high school					0.224
No	106 (40.8%)	34 (38.6%)	29 (35.4%)	43 (47.8%)	
Yes	154 (59.2%)	54 (61.4%)	53 (64.6%)	47 (52.2%)	
Marital status					<0.001
Married	158 (60.8%)	56 (63.6%)	34 (41.5%)	68 (75.6%)	
Single	102 (39.2%)	32 (36.4%)	48 (58.5%)	22 (24.4%)	
Occupation					0.006
Student or employed	197 (75.8%)	70 (79.5%)	69 (84.1%)	58 (64.4%)	
Unemployed or retired	63 (24.2%)	18 (20.5%)	13 (15.9%)	32 (35.6%)	
Insured					<0.001
No	89 (34.2%)	40 (45.5%)	39 (47.6%)	10 (11.1%)	
Yes	171 (65.8%)	48 (54.5%)	43 (52.4%)	80 (88.9%)	
Reimbursement policy covered					<0.001
No	114 (43.8%)	52 (59.1%)	45 (54.9%)	17 (18.9%)	
Yes	146 (56.2%)	36 (40.9%)	37 (45.1%)	73 (81.1%)	
Number of family members	2 (1, 3)	1 (0, 2.25)	2 (1, 3)	2 (1, 3)	0.014
Family income last year (CNY ¥)	100,000 (60,000, 150,000)	100,000 (52,000, 180,000)	90,000 (60,000, 138,750)	100,000 (60,000, 157,500)	0.576
Hypertension					0.049
No	235 (90.4%)	81 (92.0%)	78 (95.1%)	76 (84.4%)	
Yes	25 (9.6%)	7 (8.0%)	4 (4.9%)	14 (15.6%)	
Type 2 diabetes					0.610
No	248 (95.4%)	85 (96.6%)	79 (96.3%)	84 (93.3%)	
Yes	12 (4.6%)	3 (3.4%)	3 (3.7%)	6 (6.7%)	
Cavity					0.631
No	224 (86.2%)	74 (84.1%)	70 (85.4%)	80 (88.9%)	
Yes	36 (13.8%)	14 (15.9%)	12 (14.6%)	10 (11.1%)	
Combined with EPTB					0.090
No	222 (85.4%)	75 (85.2%)	65 (79.3%)	82 (91.1%)	
Yes	38 (14.6%)	13 (14.8%)	17 (20.7%)	8 (8.9%)	
Bacteriologically confirmed					0.002
No	102 (39.2%)	37 (42.0%)	42 (51.2%)	23 (25.6%)	
Yes	158 (60.8%)	51 (58.0%)	40 (48.8%)	67 (74.4%)	

Data are *n* (%) or median (IQR).

EPTB, extrapulmonary TB.

a *p*-values were for comparison between three groups using Pearson’s Chi-squared test, Fisher’s exact test, or Kruskal-Wallis rank-sum test.

### Dose intake in the mHealth reminder groups

**Figure 3:**
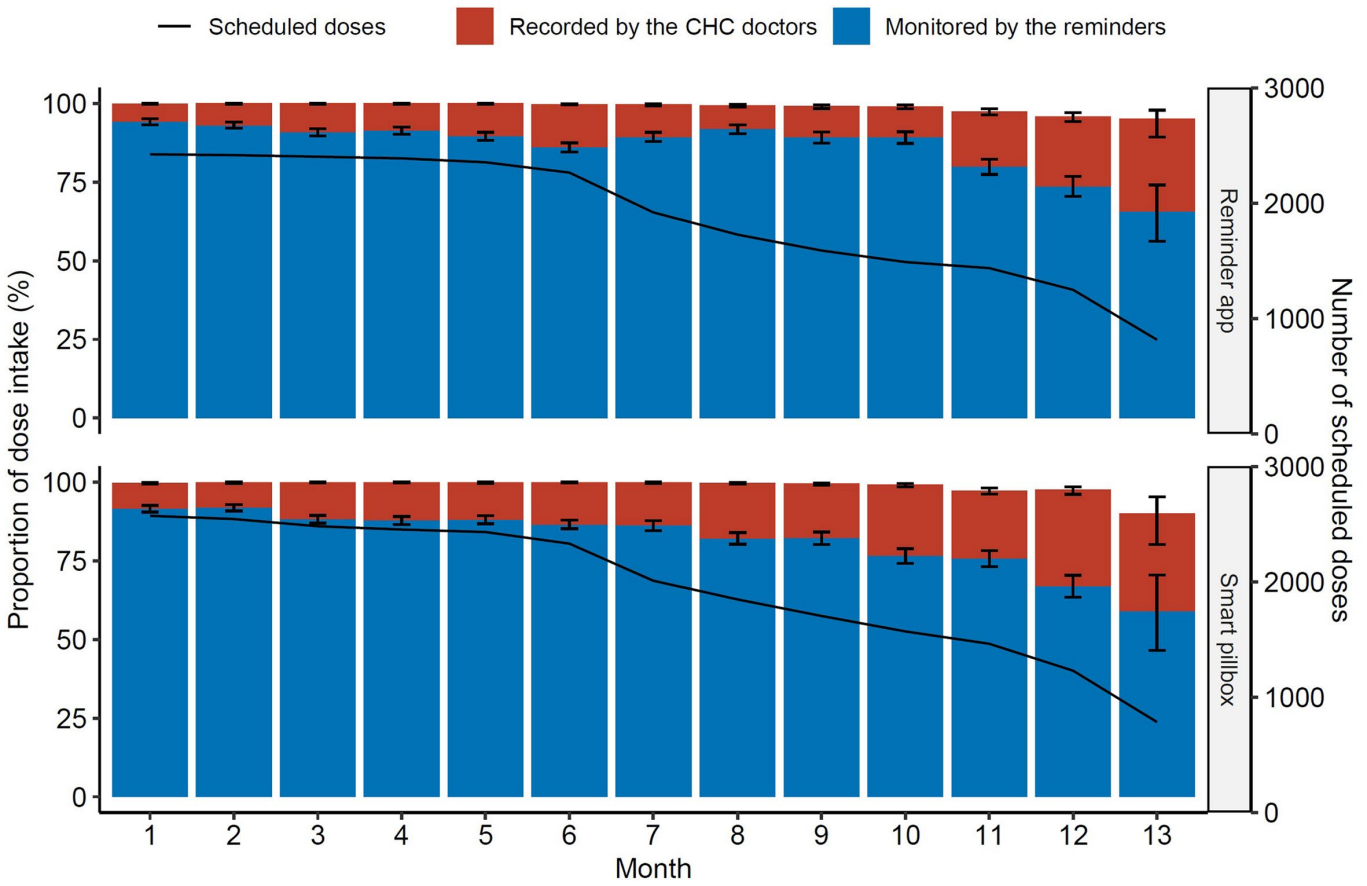
Proportions of dose intake through treatment. The black solid line indicated the monthly number of the scheduled doses. CHC, community health center.

A total of 44,785 doses were scheduled for 172 patients in the mHealth reminder groups during the study period. 44,604 (99.6%) doses were taken with 39,280 (87.7%) monitored by the mHealth reminders and 5,324 (11.9%) recorded by the CHC doctors after intervention and confirmation. Additionally, the proportion of dose intake monitored by the reminder app and the smart pillbox was 89.5 and 86.0%, respectively (*p* < 0.001). Both had a significant time-dependent downward linear trend (*p* < 0.001, Cochran-Armitage trend test, compared with month 1), ranging from 94.2 to 65.7% and from 91.6 to 59.0%, respectively (Figure 3).

Error bars were 95% CIs. The line indicated the monthly scheduled doses for all the patients.

### Effect of mHealth reminders on tuberculosis treatment outcomes

247 (95%) patients were successfully treated and 5 (2.0%) relapsed within 1 year. The treatment success rate ranged from 93.3% in the smart pillbox group to 96.6% in the standard care group, varying insignificantly between the three groups. The recurrence rate also differed insignificantly (Table 2).

**Table 2:** Treatment outcomes of patients in the study.

Outcomes	Overall (*n* = 260)	Standard care (*n* = 88)	Reminder app (*n* = 82)	Smart pillbox (*n* = 90)	*p*-value^a^
Follow-up time (person-months)	2,581	949	785	847	
Treatment success					0.599
No	13 (5.0%)	3 (3.4%)	4 (4.9%)	6 (6.7%)	
Yes	247 (95.0%)	85 (96.6%)	78 (95.1%)	84 (93.3%)	
Treatment outcomes					0.022
Treatment complete	176 (67.7%)	66 (75.0%)	62 (75.6%)	48 (53.3%)	
Cured	71 (27.3%)	19 (21.6%)	16 (19.5%)	36 (40.0%)	
Died	4 (1.5%)	1 (1.1%)	1 (1.2%)	2 (2.2%)	
Transferred out	8 (3.1%)	2 (2.3%)	3 (3.7%)	3 (3.3%)	
Treatment failed	1 (0.4%)	0 (0.0%)	0 (0.0%)	1 (1.1%)	
Recurrence					0.330
No	242 (98.0%)	83 (97.6%)	78 (100.0%)	81 (96.4%)	
Yes	5 (2.0%)	2 (2.4%)	0 (0.0%)	3 (3.6%)	

Data are *n* (%).

a *p*-values were for comparison between three groups using Pearson’s Chi-squared test or Fisher’s exact test.

**Figure 4:**
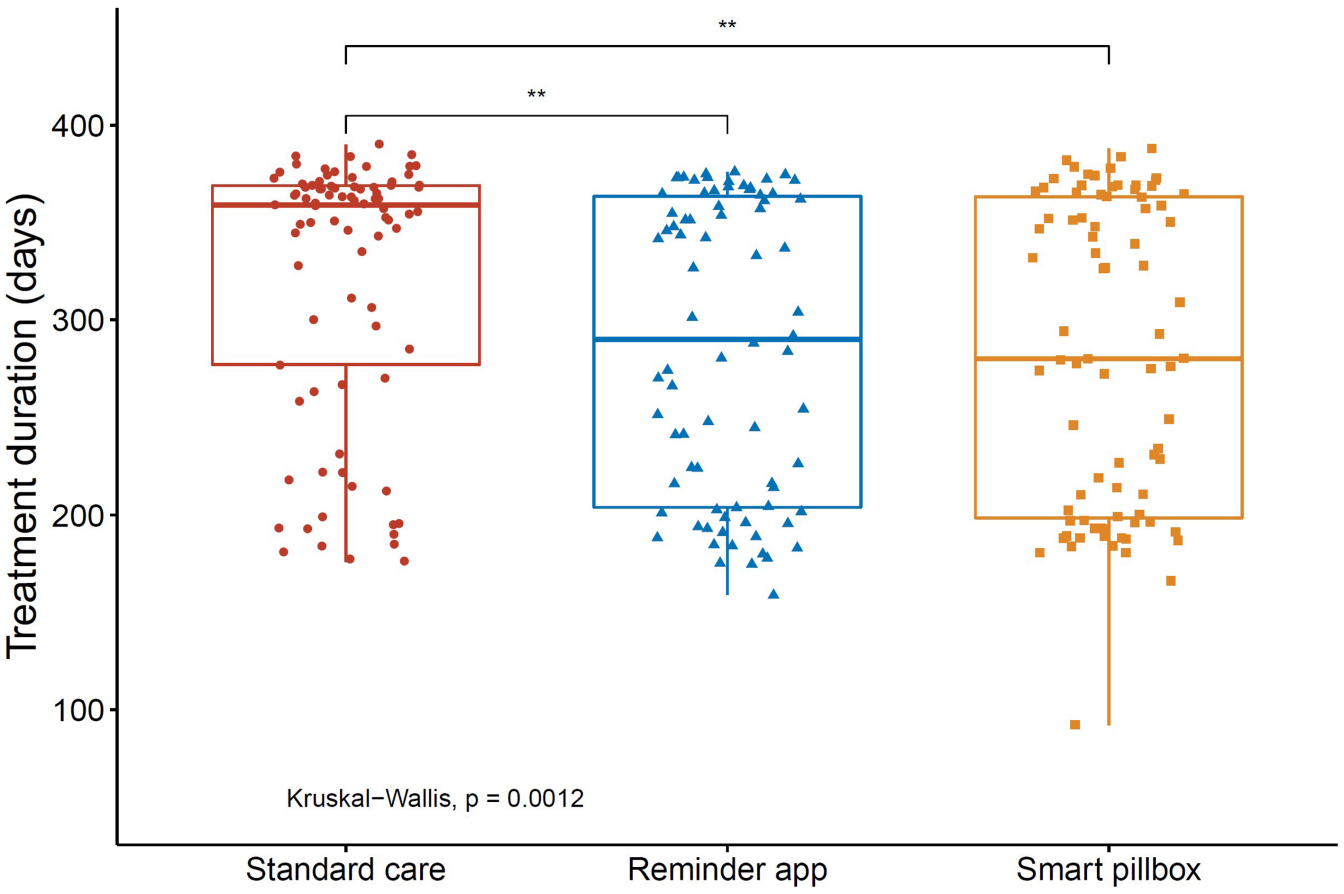
Treatment duration for successfully treated patients in the study. **p < 0.01. Treatment duration was defined as the time from the initial to the end of the anti-TB chemotherapy.

**Figure 5:**
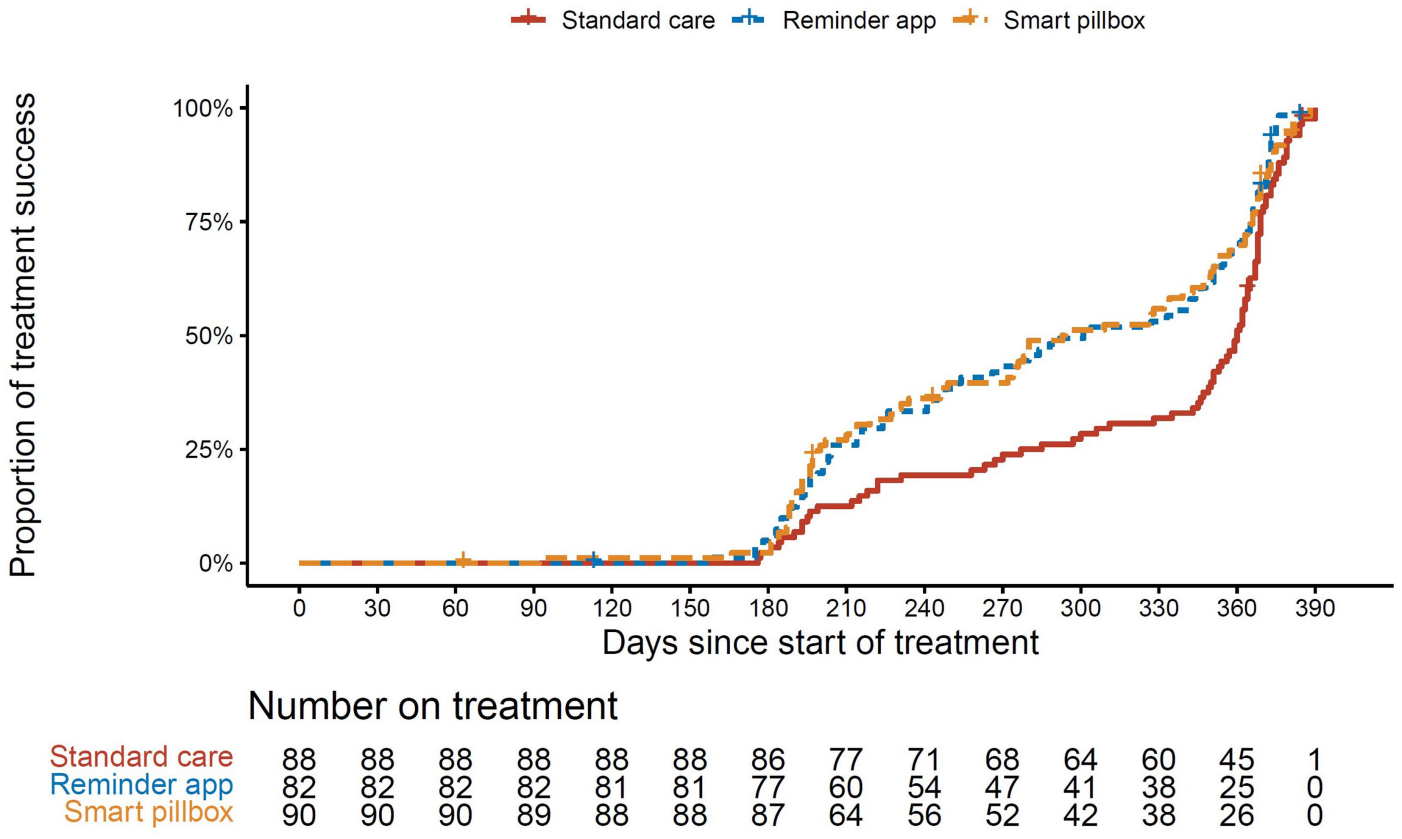
Kaplan–Meier survival estimates for treatment success.

However, we found that the treatment duration of successfully treated patients in the standard care group (median 360, IQR 283–369, mean 322, days) was significantly longer than those in the reminder app group (median 296, IQR 204–365, mean 286, days) and the smart pillbox group (median 280, IQR 198–365, mean 283, days) (both *p* < 0.01, Dunn’s test, Figure 4). The Kaplan–Meier estimates also showed that the treatment success occurred sooner in the reminder app and smart pillbox groups than in the standard care group (*p* = 0.02 and 0.03, respectively, Log-rank test adjusted by Bonferroni-Holm method, Figure 5).

In the multivariate analysis, using the reminder app to support anti-TB treatment was associated with a 1.58 times increase in the possibility of treatment success compared with the standard care (adjusted HR = 1.58, 95%CI 1.12–2.22, *p* < 0.01). Similarly, the smart pillbox was associated with a 1.63 times increase (adjusted HR = 1.63, 95%CI 1.16–2.30, *p* < 0.01). In addition, bacteriologically confirmed and extrapulmonary TB were independently associated with decreased possibility of treatment success (*p* < 0.05, Table 3). The power for the comparison of survival curves under the Cox proportional hazards model was 0.84 (the reminder app group vs. standard care group), and 0.89 (the smart pillbox group vs. standard care group), respectively.

**Table 3:** Univariate and multivariate analysis of factors associated with treatment success.

Characteristic	Univariate analysis		Multivariate analysis	
	Hazard ratio (95% CI)	*p*-value	Hazard ratio (95% CI)^a^	*p*-value
Sex				
Female	1.00	–	1.00	–
Male	0.95 (0.73, 1.24)	0.710	0.89 (0.67, 1.17)	0.4
Age, years				
18–29	1.00	–	1.00	–
30–44	0.69 (0.51, 0.95)	0.023	0.62 (0.44, 0.88)	0.007
45–59	0.98 (0.66, 1.47)	0.936	0.94 (0.61, 1.44)	0.8
> = 60	0.87 (0.61, 1.24)	0.437	0.91 (0.59, 1.40)	0.7
Migrant				
No	1.00	–		
Yes	1.09 (0.83, 1.43)	0.542		
Completed high school				
No	1.00	–		
Yes	0.90 (0.70, 1.16)	0.428		
Marital status				
Married	1.00	–		
Single	1.16 (0.90, 1.50)	0.261		
Occupation				
Student or employed	1.00	–		
Unemployed or retired	1.04 (0.77, 1.40)	0.788		
Insured				
No	1.00	–		
Yes	1.00 (0.77, 1.29)	0.975		
Reimbursement policy covered				
No	1.00	–	1.00	–
Yes	1.16 (0.90, 1.49)	0.263	1.10 (0.82, 1.48)	0.5
Number of family members	0.96 (0.88, 1.05)	0.347		
Bacteriologically confirmed				
No	1.00	–	1.00	–
Yes	0.82 (0.63, 1.06)	0.128	0.75 (0.57, 0.99)	0.044
Cavity				
No	1.00	–	1.00	–
Yes	0.71 (0.49, 1.03)	0.069	0.68 (0.46, 1.00)	0.050
Combined with EPTB				
No	1.00	–	1.00	–
Yes	0.61 (0.43, 0.87)	0.006	0.51 (0.35, 0.74)	<0.001
Type 2 diabetes				
No	1.00	–	1.00	–
Yes	1.05 (0.58, 1.93)	0.863	1.02 (0.54, 1.91)	>0.9
Group				
Standard care	1.00	–	1.00	–
Reminder app	1.50 (1.10, 2.06)	0.010	1.58 (1.12, 2.22)	0.009
Smart pillbox	1.42 (1.05, 1.93)	0.023	1.63 (1.16, 2.30)	0.005

a Adjusted for potentially confounding factors of sex, age, reimbursement policy coverage, bacteriological confirmation, cavity, combined with EPTB, and type 2 diabetes comorbidity.

EPTB, extrapulmonary TB.

## Discussion

In this prospective cohort study, we observed that both the reminder app and the smart pillbox increased the possibility of treatment success compared with the standard care under the programmatic setting in a high TB burden country (adjusted HR = 1.58 and 1.63, respectively, both *p* < 0.01). Although the treatment success rates differed insignificantly between the three groups, patients using mHealth reminders acquired treatment success much sooner than those under standard care did (both *p* < 0.05). Besides, more than 92% of participants in the mHealth reminder groups completed study periods. The dose intake rates were extremely high in two mHealth reminder groups with more than 85% of doses monitored by the reminders. Our findings suggest the acceptability of the reminder app and the smart pillbox and also the effectiveness on the TB treatment outcomes in Shanghai, China.

Smartphone apps have been widely used (22) to support the treatment of chronic diseases, such as hypertension (23) and diabetes (24). However, the apps providing drug intake reminders, follow-up alerts, and side effects monitoring to support active TB patients’ treatment are limited (25) and most assessed the effect on adherence instead of the treatment outcomes. In Shenzhen, China, a comprehensive app that mainly focused on video-observed therapy was reportedly easy-to-use and significantly increased patient adherence (26). Li et al. reported that a similar reminder app improved TB patients’ revisit examination adherence in Tianjin, China (27). Essentially, the reminder app used in the current study offered an SMS-like intervention as described by WHO (9). The effect of SMS on TB treatment adherence and success was controversial in the trial settings (4, 28). But in our cohort, the reminder app was associated with a 1.58 times increase in the possibility of treatment success under the programmatic setting. This might result from the additional interactive services such as the patient forum, and instant communication with CHC doctors.

In China, an EMM that did not provide real-time data was proved to be beneficial to improving TB patients’ adherence in a randomized trial (11). Moreover, a recent ecological study suggested that it also improved the treatment outcomes under programmatic conditions (10). In our cohort, we observed a similar result that the smart pillbox was associated with a 1.63 times increase in the possibility of treatment success under the programmatic setting. This was also consistent with other real-time EMM studies in Morocco (17) and South Africa (29). Real-time data transport is the foundation to enable instant interaction between patients and healthcare providers. China has a strong and widely covered national telecommunication network, which makes it feasible to scale up the application of real-time EMMs to provide better care for TB patients.

In our cohort, all of the treatment success rates across the three groups surpassed 90%. Meanwhile, the treatment duration was extremely long compared with the six-month standard regimen. Although the 2HREZ/4HR regimen was universally adopted at the start of treatment for new drug-susceptible TB patients in Shanghai, it was very common to extend the continuous phase of anti-TB therapy if the treatment effect was not satisfying (30). We observed that the median treatment duration of successfully treated patients in two mHealth reminder groups was significantly less than that in the standard care group (both *p* < 0.05), which indicated that the patients acquired treatment success much sooner and had a lower drug burden. Therefore, we used the Cox proportional hazard model to take into account the treatment duration and adjust other potentially associated factors.

The overall proportions of dose intake monitored by the reminder app (89.5%) and the smart pillbox (86.0%) in our study were slightly higher than the results from the studies using similar interventions in Tianjin, China (84.8%) (27) and Morocco (81.3%) (17). However, the decreasing trend over the months was similar. This might indicate the need to provide more care for patients while the treatment duration was extended.

WHO has suggested that the treatment adherence interventions be selected based on the individual patient’s needs (2). In this study, the mHealth reminder system offered two interventions and participants chose the study group voluntarily. We found the patients in the smart pillbox group were significantly older than those in the reminder app group. Although the smart pillbox is almost maintenance-free except for recharging, young people are familiar with using smartphone apps and have more outdoor activities, they might feel uncomfortable carrying the pillbox with them. It is worth mentioning that the mHealth reminder system is open-ended and can be connected to more reminders such as the bracelet (31) and the smaller pillbox in the future to meet more patients’ needs.

To our knowledge, this was the first study to assess the effectiveness of the reminder app and the smart pillbox that was integrated into one system on TB treatment outcomes globally. There were still several limitations in our study. Firstly, the observational study design reflected the real-world situation and provided suggestions for the integration of mHealth reminders into the TB program, but made it impossible to randomize the participants at enrollment. Although the multivariate model has been adopted to adjust potential confounders, the difference in treatment outcomes could still be partially attributed to imbalanced factors. In the future, randomized clinical trials (RCTs) (32, 33) are needed to provide a stricter evaluation of the effectiveness. Secondly, to make the treatment outcomes comparable between study groups, we excluded previously treated and rifampicin-resistant patients from our study as several RCTs (32, 34) did. However, these patients usually have more lengthy treatments and face more problems with treatment adherence (3). Therefore, applying mHealth interventions to them should be cautious before more evidence emerge. Lastly, the drug intake data collected through mHealth reminders was indirect evidence of adherence and there was no verification of the data using methods such as drug urine tests. But one study (35) comparing EMM data and urine tests for traces of TB drugs proved the high correlation between reminders data and adherence.

## Conclusion

In conclusion, the reminder app and the smart pillbox interventions were acceptable and improved the treatment outcomes compared with the standard care under the programmatic setting in Shanghai, China. More high-level evidence is expected to confirm the effect of mHealth reminders on TB treatment outcomes.

## Data availability statement

The original contributions presented in the study are included in the article/Supplementary material, further inquiries can be directed to the corresponding authors.

## Ethics statement

The studies involving human participants were reviewed and approved by the Ethical Review Committee at Shanghai Municipal Center for Disease Control and Prevention (2019-14). The patients/participants provided their written informed consent to participate in this study.

## Author contributions

ZW and LL participated in the study design, data collecting, and statistical analysis. YL, ZZ, and CN participated in the data collecting and statistical analysis. ZW wrote the first draft of the manuscript. JC, QP, and ZY reviewed and revised the first draft. XS and WZ conceived, designed, and managed the study. All authors contributed to the article and approved the submitted version.

## Funding

The study was funded by the Chinese National Science and Technology Major Project (2018ZX10715012), Shanghai Municipal Health Commission (20204Y0212), Shanghai “Rising Stars of Medical Talent” Youth Development Program, the Shanghai Municipal Project for Academic Leaders in Public Health (GWV-10.2-XD23), and Songjiang Science and Technology Key Project (20SJKJGG206). The sponsors of the study had no role in study design, data collection, data analysis, data interpretation, or writing of the manuscript.

## Conflict of interest

The authors declare that the research was conducted in the absence of any commercial or financial relationships that could be construed as a potential conflict of interest.

The reviewer QZ declared a shared affiliation with the author ZW to the handling editor at the time of review.

## Publisher’s note

All claims expressed in this article are solely those of the authors and do not necessarily represent those of their affiliated organizations, or those of the publisher, the editors and the reviewers. Any product that may be evaluated in this article, or claim that may be made by its manufacturer, is not guaranteed or endorsed by the publisher.

## Figures and Tables

**Table:** 

app	application
CDC	Center for Diseases Control and Prevention
CHC	community health center
DOT	directly observed therapy
EMM	electronic medication monitor
EPTB	extrapulmonary TB
IQR	interquartile range
MEMS	medication event monitoring system
mHealth	mobile health
PTB	pulmonary TB
RCT	randomized clinical trial
SAT	self-administered therapy
SIM	subscriber identity module
SMS	short message service
TB	tuberculosis
TBMIS	TB management information system
VOT	video-supported treatment
WHO	World Health Organization
