# Supplementary material for: Effect of mobile health reminders on tuberculosis treatment outcomes in Shanghai, China: A prospective cohort study
Source: Front Public Health. 2023 Apr 27;11:923319. doi: 10.3389/fpubh.2023.923319 (PMC10173861; doi:10.3389/fpubh.2023.923319)

**Supplementary Material**

The interface of the reminder app.


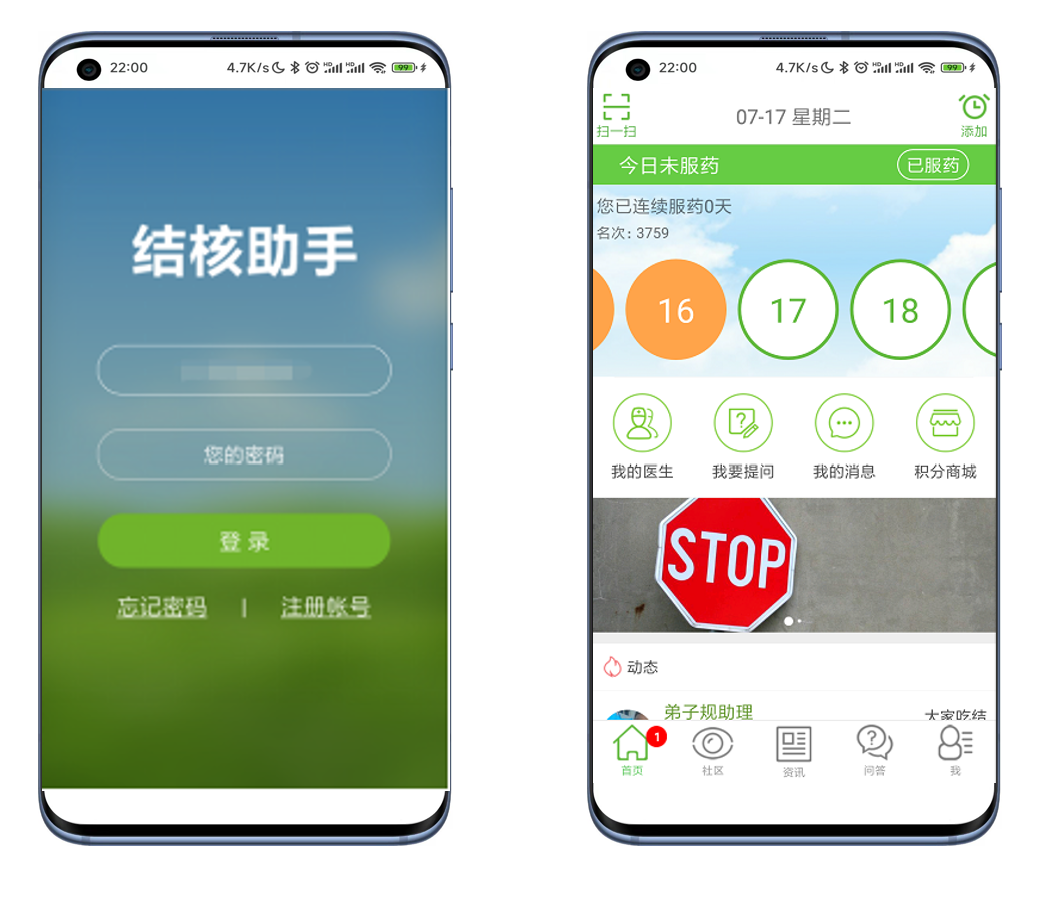


The appearance of the smart pillbox.


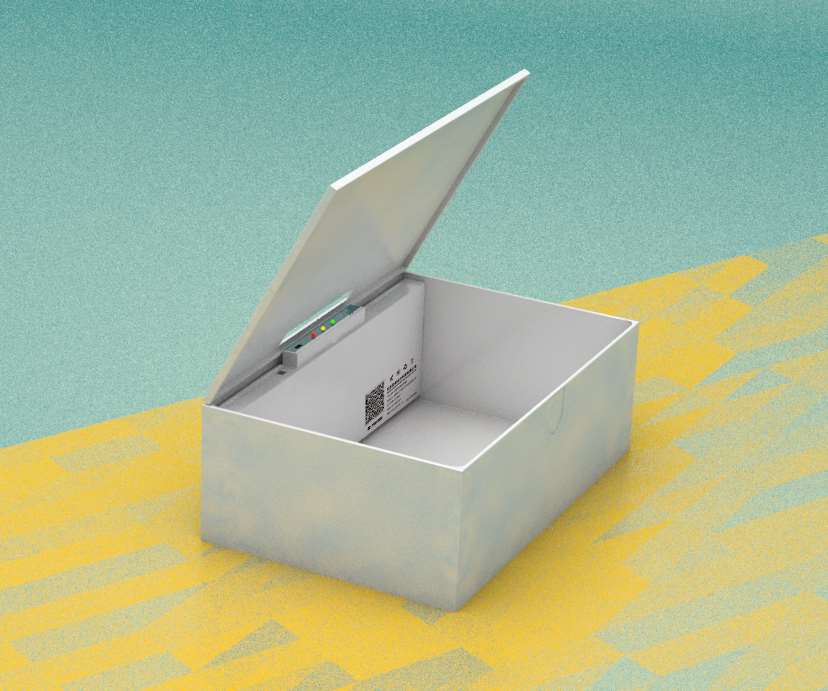


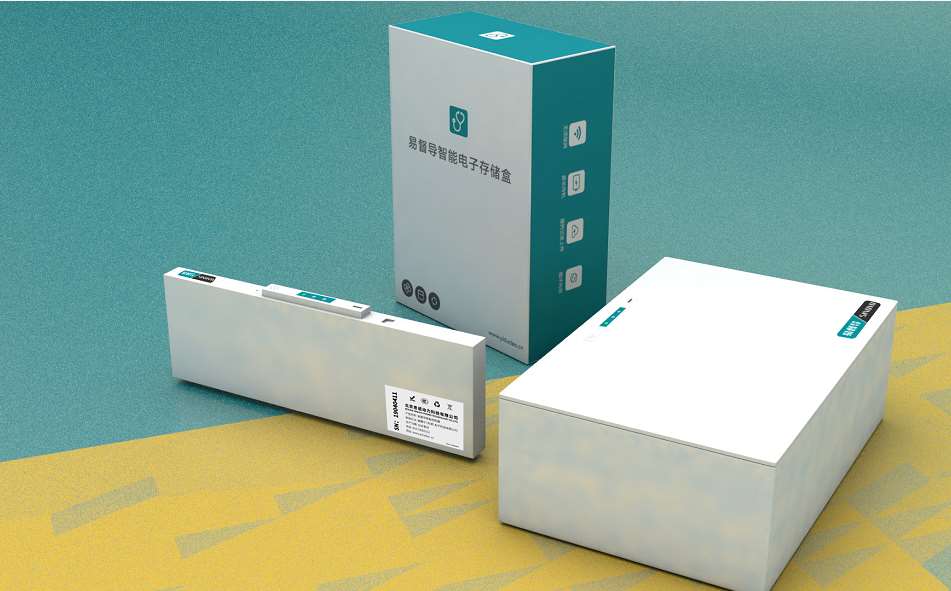


The community health center doctor introduced the usage of the smart pillbox to tuberculosis patients in Shanghai, China.


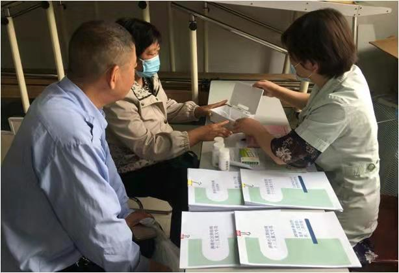

Supplement: Supplementary file 1 [file Data_Sheet_1.docx]
